# RHDV2 epidemic in UK pet rabbits. Part 1: clinical features, gross *post mortem* and histopathological findings

**DOI:** 10.1111/jsap.13141

**Published:** 2020-05-08

**Authors:** N. Harcourt‐Brown, M. Silkstone, T. J. Whitbread, F. M. Harcourt‐Brown

**Affiliations:** ^1^ North Yorkshire UK; ^2^ Abbey Veterinary Services Newton Abbot UK; ^3^ Abbey Veterinary Services Newton Abbot UK

## Abstract

**Objectives:**

To report clinical features, gross *post mortem* and histopathological findings from an investigation into sudden or unexpected death in rabbits that was undertaken during an outbreak of rabbit haemorrhagic disease.

**Materials and Methods:**

Using a standard protocol, veterinarians were invited to submit case histories and results of their *post mortem* examination of pet rabbits that died unexpectedly. Histopathological examination of heart, lungs, liver, spleen and kidney samples was collated with macroscopic appearance and clinical details.

**Results:**

Hepatocellular necrosis, characteristic of rabbit haemorrhagic disease, was observed in 185 of 300 (62%) submissions, often accompanied by glomerular thrombosis and changes in other organs. Evidence of rabbit haemorrhagic disease was not apparent on histopathology in 113 of 300 (38%) rabbits. Gross *post mortem* examination by veterinary practitioners did not always reflect reported histopathological changes. No macroscopic abnormalities were seen in 78/185 (42%) of rabbit haemorrhagic disease cases. Rapid death and death of other rabbits in the household were common features of rabbit haemorrhagic disease. *Ante mortem* clinical signs included anorexia, collapse, lethargy, seizures, icterus, bleeding from the mouth, dyspnoea, hypothermia, pyrexia, bradycardia or poor blood clotting.

**Clinical Importance:**

Rabbit haemorrhagic disease can be suspected from a history of sudden death, especially if multiple rabbits are affected. There is not always macroscopic evidence of the disease but histopathology is useful to support or refute a diagnosis of rabbit haemorrhagic disease and provide information about other causes of death.

## INTRODUCTION

Rabbit haemorrhagic disease (RHD) is a highly contagious and potentially lethal viral disease that affects wild and domestic rabbits and causes acute hepatocellular necrosis. The RHD virus (RHDV) is a calicivirus of the genus *Lagovirus* that targets hepatocytes and cells of the mononuclear phagocytic system, such as Kupffer cells, circulating monocytes, alveolar macrophages and endothelial cells (Ramiro‐Ibáñez *et al*. 1999, Neimanis *et al*. 2018). Death is due to liver failure or disseminated intravascular coagulopathy (Marcato *et al*. 1991, Ueda *et al*. 1992). It is probable that the apoptosis of intravascular monocytes and endothelial cells is the trigger for disseminated intravascular coagulopathy (Alonso *et al*. 1998, Ramiro‐Ibáñez *et al*. 1999).

RHD was first identified in China in 1984 (Liu *et al*. 1984) and by 1988 the disease had spread throughout Europe and many other countries worldwide causing losses in the meat industry and reducing wild rabbit populations (Morisse *et al*. 1991, Villafuerte *et al*. 2013). In the UK, RHD was first reported in 1992 in a group of exhibition rabbits (Fuller *et al*. 1993) before spreading through the wild and domestic rabbit population. In 2010, a new serotype of the RHD virus (RHDV2) was identified from an outbreak of RHD in vaccinated meat rabbits in a rabbitry in France (Le Gall‐Reculé *et al*. 2011) before spreading rapidly throughout Europe (Neimanis *et al*. 2018, Silvério et al. 2018) and reaching other continents including Canada and New Zealand (OIE 2019a). RHDV2 is phylogenetically, antigenically and serotypically distinct from previously identified strains of RHDV (RHDV1) (Le Gall‐Reculé *et al*. 2013). The discovery of new strains and serotypes of *Lagovirus* has resulted in complex nomenclature and a unified classification system based on genomic characteristics of the viral strains has been proposed (Le Pendu *et al*. 2017). Under this system, the two serotypes of RHDV are named *Lagovirus europaeus GI.1* and *Lagovirus europaeus GI.2* (Le Pendu et al. 2017). From a clinician's perspective, these serotypes are usually referred to as RHDV1 and RHDV2.

RHD has been investigated extensively in the laboratory where the clinical course of the disease and the pathological changes can be monitored closely after an infective dose of the virus (Ueda *et al*. 1992, Lavazza *et al*. 1996, Alonso *et al*. 1998, Mikami *et al*. 1999, Prieto *et al*. 2000, Kimura *et al*. 2001, Chen *et al*. 2008, Le Gall‐Reculé *et al*. 2013, Capucci *et al*. 2017, Dalton *et al*. 2018, Neimanis *et al*. 2018, Le Minor *et al*. 2019). The clinical course of RHD has been described as peracute, acute or chronic (Marcato *et al*. 1991, Abrantes *et al*. 2012, OIE 2019b). In the peracute form, rabbits die suddenly and without clinical signs. In the acute form of the disease rabbits show lethargy and pyrexia (>40°C) in conjunction with neurologic or respiratory signs. Evidence of haemorrhage may be evident as epistaxis or haematuria. In the chronic form of the disease, jaundice, anorexia and lethargy occurs and these rabbits tend to die 1 to 2 weeks later. Subacute forms of the disease can occur with rabbits showing milder clinical signs and surviving. In contrast to laboratory investigations, detailed accounts of the clinical features of RHD in naturally occurring outbreaks are rare (Soliman *et al*. 2016, Bonvehí *et al*. 2019). Although deaths from RHD are included in mortality surveys of meat rabbits, these animals had minimal observation prior to death (Rosell & de la Fuente 2009, Rosell & de la Fuente 2016)

Post mortem examination and histopathology play a major role in the diagnosis of RHD (Marcato *et al*. 1991, Chasey 1997, Abrantes *et al*. 2012, Kerr & Donnelly 2013, Barthold *et al*. 2016, Soliman *et al*. 2016, Rocchi & Dagleish 2018, OIE 2019b) and, indeed, histopathological changes have been described as pathognomonic (Fuchs & Weissenböck 1992, Neimanis 2018). Described macroscopic changes include an enlarged, pale or congested liver with a distinct lobular pattern and an enlarged spleen that is often dark and engorged. Multifocal haemorrhages may be seen in the liver and other organs such as lungs, kidney and heart. Tracheal haemorrhage can occur, and jaundice is occasionally encountered. There is acute hepatocellular necrosis often accompanied by signs of disseminated coagulopathy in other organs. Although histology can be diagnostic for RHD, further tests are necessary to demonstrate the virus and identify the strain. These include PCR, enzyme‐linked immunosorbent assay (ELISA), haemagglutination (HA) tests, electron microscopy or immunohistochemistry (Capucci *et al*. 1991, OIE 2019b). Although these techniques may be available in research laboratories, most are not available to the veterinary practitioner, especially in UK where rabbit meat is unpopular. At the time of writing, the only diagnostic tools for RHD in general practice in the UK are gross *post mortem* examination, histopathology and PCR testing for RHDV1 and RHDV2.

The clinical, gross *post mortem* and microscopic features of the rabbits in which RHD was diagnosed by histopathology are presented in this article. PCR results and vaccination status of rabbits with confirmed RHDV2 are presented in Part 2.

## MATERIALS AND METHODS

From November 2016 to December 2018, an investigation into sudden and unexpected death in pet rabbits was conducted. Although the investigation was not aimed at RHD, it coincided with an epidemic of RHDV2 in the UK and provided observational information about a naturally‐occurring outbreak of RHDV2. The investigation was advertised on a website. Histopathology and, if applicable, PCR testing were offered free of charge to participating veterinary practitioners who performed *post mortem* examinations on rabbits that died suddenly or unexpectedly. The investigation was limited to rabbits kept as pets and included anaesthetic deaths, rabbits that were found dead or dying at home and those that died during hospitalisation at a veterinary practice. Collapsed rabbits that were euthanased were also included. Informed consent to the *post mortem* examination was given by each owner. The investigation was approved by the Ethics Review Panel at the Royal College of Veterinary Surgeons.

A non‐forensic *post mortem* examination was performed by the veterinary practitioner within 6 hours of death and followed a protocol we recommended. This began by inspecting the carcase for signs of trauma before examining the oral cavity, internal organs and body cavities. The spleen, a kidney and the pluck (trachea, lungs, heart and, if present, thymus) were collected. Before removing the pluck, the trachea was opened to the level of the pharynx to check for respiratory tract obstruction. Formol saline was instilled into the lungs *via* the trachea. Representative samples of liver were also collected. One or more liver samples were frozen, and another liver sample was fixed in formol saline along with the pluck, a kidney and the spleen. If the practitioner was interested in other tissues, additional samples were collected, fixed and included in the histopathological examination. Details of the age, sex and breed of rabbit and a description of the clinical history and *post mortem* findings were entered on an online submission form, which was enclosed with the fixed samples that were sent to a specified laboratory for histopathological examination by one of five pathologists.

At the laboratory, the tissues were processed routinely for microscopic examination. Deparaffinised sections of 3 to 4 μm were stained with haematoxylin and eosin.

Copies of the online submission form were visible to the authors that were conducting the sudden and unexpected death investigation. The histopathology results were sent to both the submitting veterinary surgeon and to us.

Samples of frozen liver were sent for PCR testing from 195 rabbits (Part 2). At the end of the study, seven cases with conflicting results between histopathology and PCR test results were reviewed histopathologically. New tissue sections were cut, stained and examined by two pathologists who were blinded to the original histopathology report, PCR results or each other's assessment.

## RESULTS

**Figure 1:**
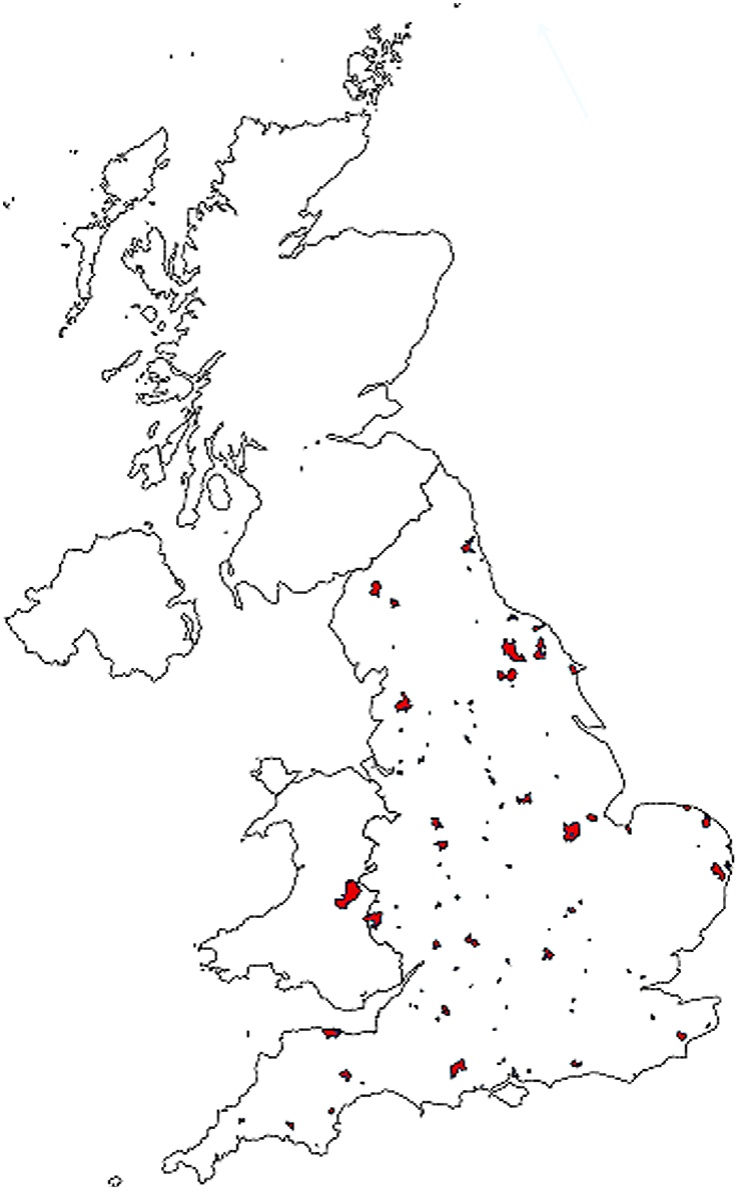
Distribution of practices that submitted tissues from 185 rabbits with characteristic histopathological features of rabbit haemorrhagic disease

Case histories, macroscopic findings and histopathology results were available from 300 rabbits that died suddenly. Of these, 185 of 300 rabbits showed characteristic histopathological features of RHD. Submissions from these 185 rabbits came from 131 practices, predominantly in England, with a few submissions from Wales and Scotland (Fig. 1).

### Histopathological features of RHD

**Figure 2:**
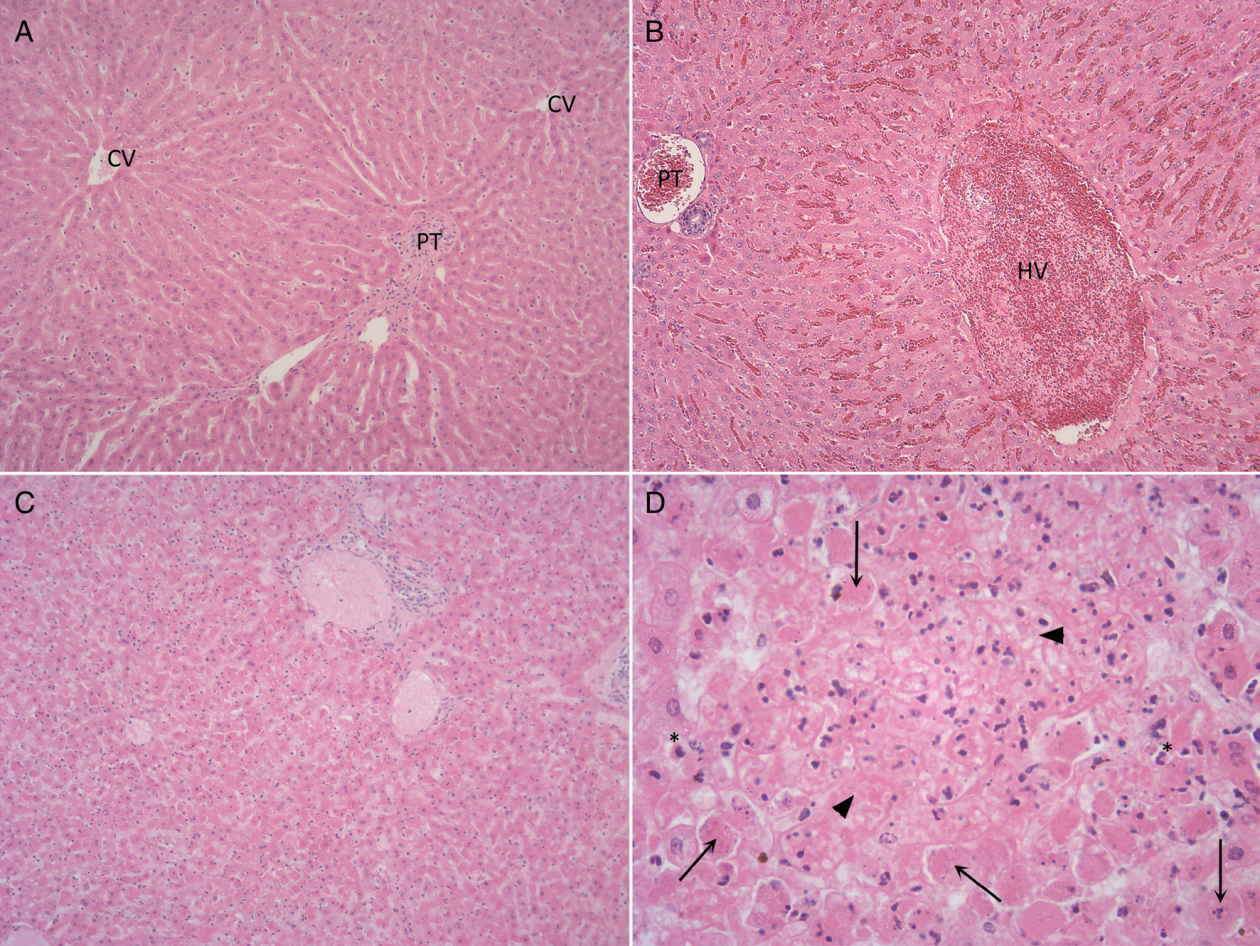
Histopathological feature of hepatocellular necrosis associated with rabbit haemorrhagic disease (RHD). (A) Normal liver (x100): CV: Central vein, PT: Portal tract. (B) Normal liver (x100): Liver of unaffected rabbit, congestion of sinusoids and hepatic vein (HV)‐ a frequent terminal event without necrosis and inflammation. Portal tract (PT). (C) RHD (x100): Normal sinusoidal architecture disrupted by widespread hepatocellular necrosis. (D) RHD (x400): Acute necrosis with individualised necrotic hepatocytes (arrow) hepatocellular loss, deposition of fibrin (arrow head) and mild infiltration of heterophils

**Figure 3:**
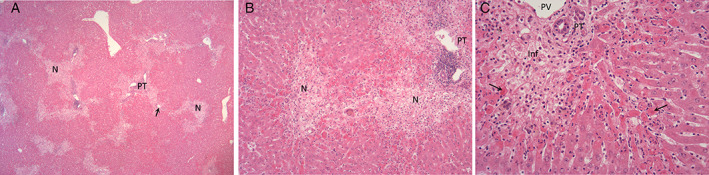
Subacute severe hepatocellular necrosis associated with rabbit haemorrhagic disease. (A) (x25) (B) (x100) (C) (x200) Subacute periportal to midzonal necrosis (N) with hepatocellular loss, active necrosis (arrow) and mild infiltration of heterophils. PT: Portal tract, N: Necrosis, PV: Portal vein, Inf: Inflammation

**Figure 4:**
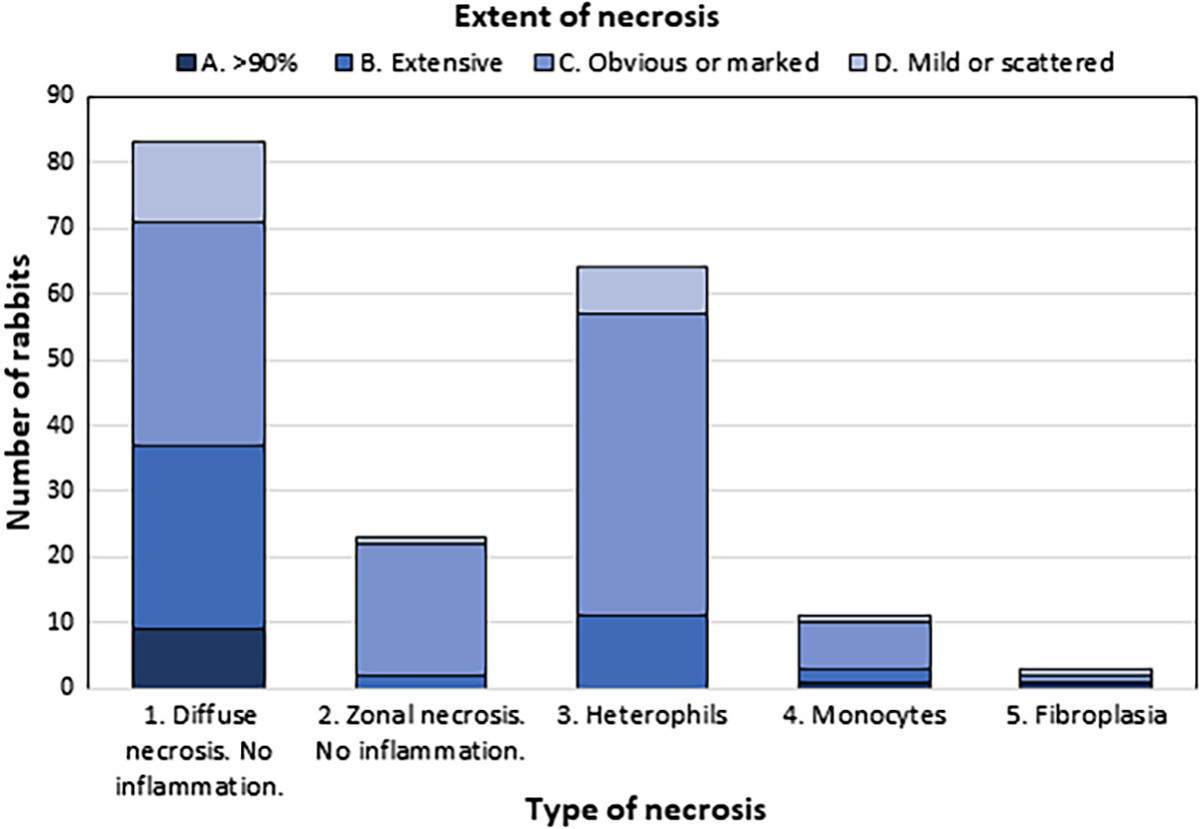
Type and extent of hepatocellular necrosis in 185 rabbits with histopathological features of rabbit haemorrhagic disease (RHD). This graph is from data extracted from histology reports from the 185 rabbits with histopathological features of RHD. All showed coagulative hepatocellular necrosis. The graph shows the degree of necrosis and the length of time that it was present before the rabbit died, which was based on inflammatory cell succession

**Figure 5:**
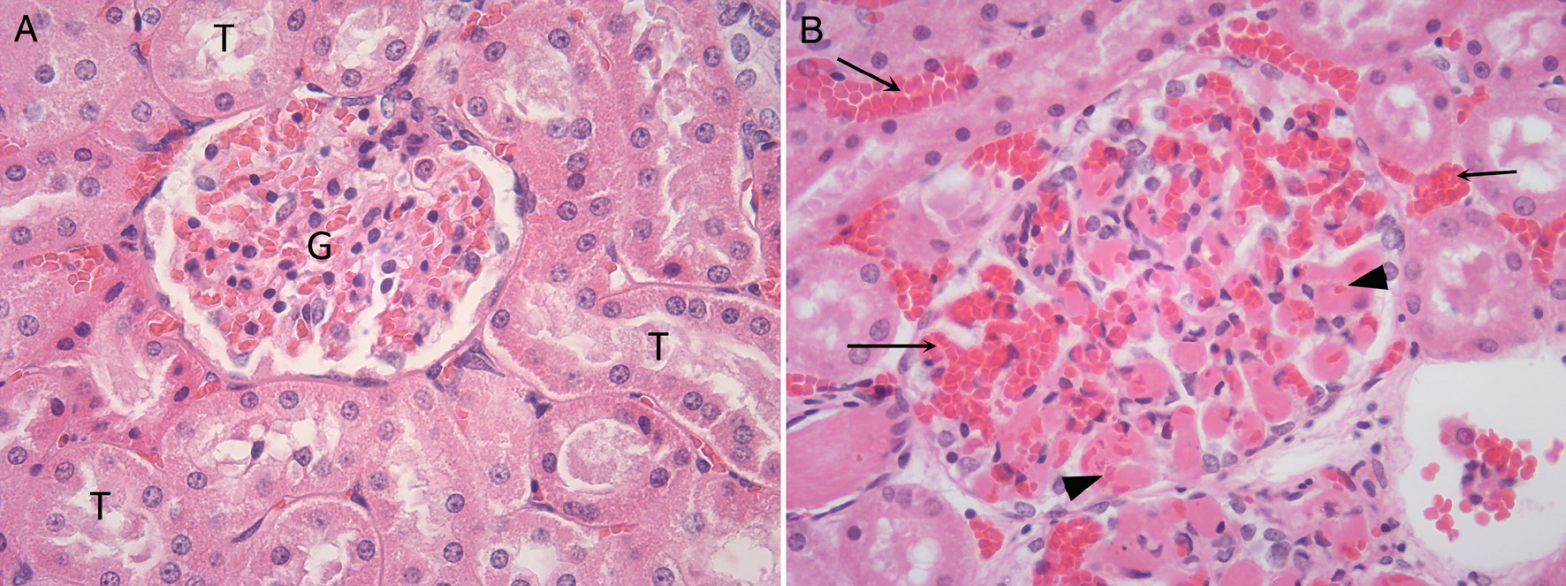
Changes in the kidney associated with rabbit haemorrhagic disease. (A) (x400): Unaffected rabbit: mild congestion of glomerulus. G: Glomerulus, T: Renal tubule. (B) RHD (x400): Affected rabbit: marked congestion (arrow) and thrombosed (arrow head) glomerular capillaries

The characteristic histopathological features of RHD included apoptosis and variable lytic and coagulative hepatocellular necrosis with a periportal to multifocal to diffuse distribution. The necrosis was sometimes accompanied by variable congestion, haemorrhage and sinusoidal fibrin thrombi (Figs. 2 and 3). Inflammatory infiltrates, of heterophils and fewer macrophages, were generally mild but more marked in less acute cases and in cases showing larger foci of confluent necrotic hepatocytes. The type and distribution of necrosis is shown in Fig. 4. Extra‐hepatic changes included fibrin thrombi, particularly in the glomeruli of kidneys (Fig. 5) and lungs. Haemorrhages were seen in various organs. Lymphocytolysis, deposition of fibrin and haemorrhages were sometimes evident in the spleen. The number of cases showing these microscopic changes is shown in Table 1.

**Table 1:** Prevalence of microscopic lesions in 185 rabbits with histopathological features of rabbit haemorrhagic disease

Organ	Number of cases in which organ was examined	Number of cases showing histopathological change	Percentage of organs examined showing histopathological change
Liver	185		
Hepatocellular necrosis		185	100
Haemorrhages		4	2.2
Kidney	185		
Glomerular thrombosis		102	55.1
Spleen	185		
Fibrin in red pulp		49	26.5
Lymphocytolysis		19	10.3
Haemorrhages		19	10.3
Heart	185		
Myocardial haemorrhages		9	4.9
Lung	185		
Alveolar haemorrhages		33	17.8
Thrombosis		17	9.2
Thymus	92		
Haemorrhages		9	9.8

**Figure 6:**
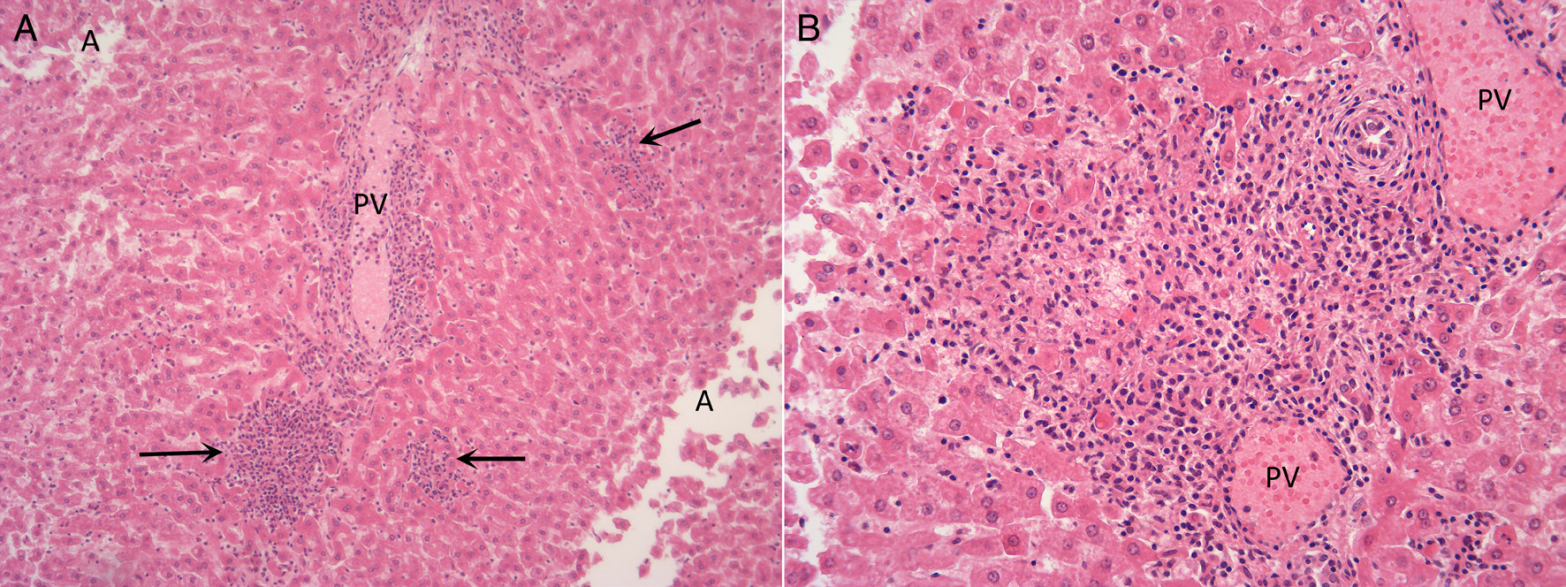
Equivocal case: (A) (x100) Foci of necrosis and inflammatory cell infiltrates (arrow) in periportal regions. PV: Portal vein, A: Artefact. (B) (x200): Recently necrotic, hypereosinophilic hepatocytes, loss of hepatocytes and infiltration of mononuclear cells and fewer heterophils. PV: Portal vein

There were two cases that showed histopathological changes that were suspicious for, but not characteristic of, RHD. In the first case, there was some focal parenchymal inflammation and cell degeneration but not with the typical distribution of lesions of RHD. The areas were predominantly periportal but adjacent to the central veins and occasionally elsewhere in the parenchyma (Fig. 6). There were also a small number of dilated bile ducts containing coccidia. The second case that was classed as suspicious of RHD showed no histopathological features of RHD on initial histopathological examination, but foci of hepatocellular necrosis and glomerular thrombosis were apparent when the case was reviewed because of a positive PCR test result (Part 2).

### Other causes of death

**Figure 7:**
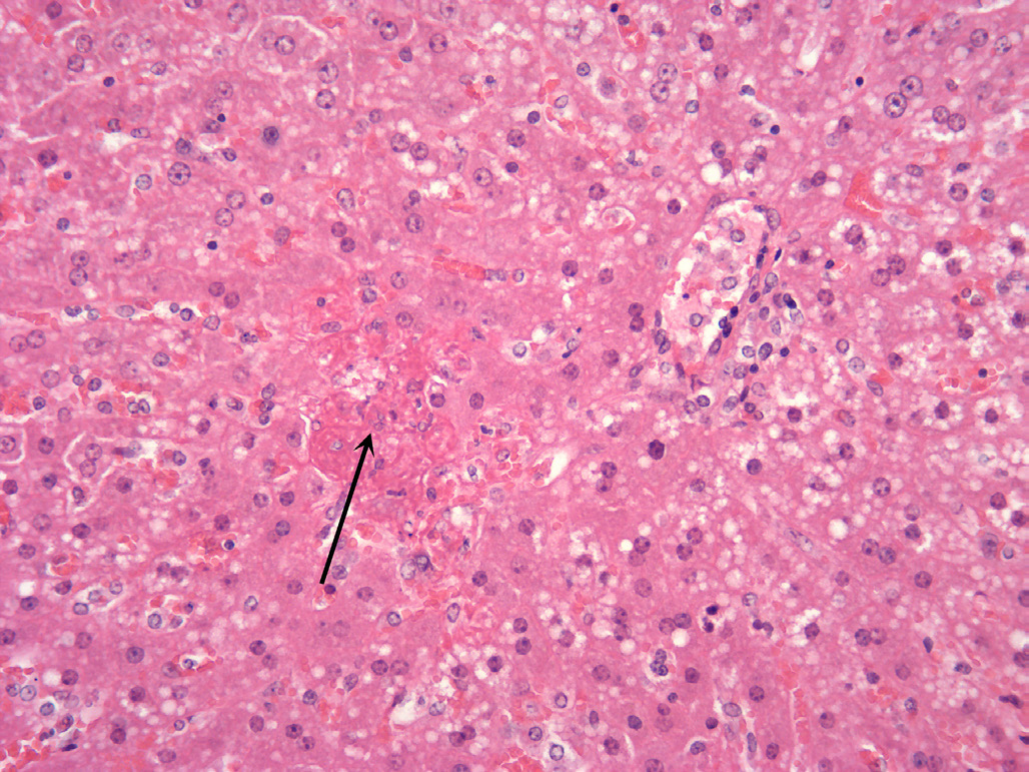
Hepatic necrosis due to sepsis. (x400): Hepatocellular vacuolation with focal lytic necrosis (arrow) and deposition of fibrin. Bacteria were noted in the capsule and septic thrombi in the lung of this animal

Histopathological features of RHD were absent in 113 of 300 rabbits in the study and the cause of death was undetermined in 35 of these 113 cases. In the remaining 78 of 113 cases, a cause of death was established from the history, gross *post mortem* findings and histopathological changes. These included respiratory disease (n = 13) including four cases of aspiration pneumonia, gastrointestinal disease (n = 14), liver disease (n = 14) including three cases of liver lobe torsion, heart disease (n = 11), renal fibrosis (n = 9), sepsis (n = 9) (Fig. 7), neoplasia (n = 3), pancreatitis (n = 2), trauma (n = 1), myxomatosis (n = 1) and necrotic tongue (n = 1).

### Clinical findings in rabbits with histopathological features of RHD

The 185 rabbits that showed histopathological features of RHD comprised 44 entire males, 40 neutered males, 64 entire females and 37 neutered females. A variety of breeds and crossbreeds were represented, and their ages ranged from 4 weeks to 7 years old with a median age of 14 months. Although the number of rabbits in the household was not specified in the clinical history, in 121 of 185 (65%) cases, death of additional rabbits was reported. In 50 of these households, both of a bonded pair of rabbits died.

“Sudden death,” with no further detail was recorded as the clinical history in 74 of 185 cases. More clinical information was available for the remaining 111 of 185 cases. Although three owners said their rabbit was “off colour” or a little subdued, most owners reported that their rabbit was eating and apparently normal when it was last seen alive. Twenty‐five rabbits were found dead in the morning after appearing to be well the night before. One rabbit was seen eating 30 minutes before death. Another died 2 hours after eating normally. Two rabbits died with food in their mouth. One rabbit had passed a health check by a veterinary surgeon 90 minutes before death. Two others died within 3 hours of a health‐check and vaccination. Twelve rabbits died on the way to the veterinary clinic. Two died in the waiting room and one in the consulting room.

Fifty‐one of the 185 rabbits with histopathological features of RHD were examined alive. Of these, nine were euthanased immediately because they were dying. In the remaining 42 cases, anorexia (n = 15), collapse (n = 16), lethargy (n = 8), seizures (n = 5), icterus (n = 5), bleeding from the mouth (n = 2), dyspnoea (n = 3), hypothermia (n = 2), pyrexia (n = 2), bradycardia (n = 1) and poor blood clotting (n = 1) were reported. Three cases were blood sampled and hypoglycaemia (n = 2) and raised liver enzymes and anaemia (n = 1) were reported. Gut stasis was diagnosed in four rabbits and received treatment. All four died shortly after leaving the veterinary clinic.

### Macroscopic findings in 185 rabbits with histopathological features of RHD

The section about gross *post mortem* findings was left blank on the submission form in nine of the 185 cases with characteristic histopathological features of RHD. “No gross abnormalities” were reported in a further 78 of 185 (42%) cases. In the 98 cases with reported gross abnormalities, a variety of changes in several permutations were recorded (Table 2). Icterus was noted in five cases. Two recorded icteric sclera and one reported icteric pinnae. The remaining two recorded icterus as a clinical sign.

**Table 2:** Macroscopic *post mortem* findings in 185 rabbits with histopathological features of rabbit haemorrhagic disease

Gross changes	Number
NOT RECORDED	8
NO OBVIOUS ABNORMALITY DETECTED	78
LIVER CHANGES Mottled liver Alone (3) **Plus** hepatomegaly (1), peritoneal fluid (5), lung haemorrhages (3), lung haemorrhages and blood at nares (1), kidney petechiation and blood at nares (1), lung haemorrhages and kidney petechiation (1), lung haemorrhages and blood in abdomen (1), lung haemorrhages and splenomegaly (1), lung haemorrhages and hepatomegaly (1), kidney petechiation and splenomegaly (1), mottled spleen (1), myocardial haemorrhage (1), blood at nares (1)	23
Haemorrhages on liver Alone (3) **Plus** lung haemorrhages (2), lung haemorrhages, hepatomegaly and splenomegaly (2) peritoneal fluid (1), splenomegaly (1), blood at nares(1), hepatomegaly and pleural haemorrhage (1), hepatomegaly and peritoneal haemorrhage (1), lung haemorrhages and peritoneal fluid (1), lung haemorrhages and blood at nares (1), lung haemorrhages, splenomegaly and myocardial haemorrhages (1)	15
Pale liver Alone (3) **Plus** haemorrhages on liver (1), lung haemorrhages (1)	5
Hepatomegaly Alone (2) **Plus** kidney petechiation (2), splenomegaly (1)	5
Miscellaneous liver changes Discoloured liver (2), discoloured liver and blood at nares (1), unspecified liver lesion (1), black spot on liver (1), congested liver (1), black patches on liver plus hepatomegaly, lung haemorrhages and blood in abdomen (1)	7
LUNG CHANGES WITHOUT LIVER ABNORMALITIES Lung haemorrhages Alone (4) **Plus** blood at nares (1), blood in pleural cavity (2), bloody peritoneal fluid (1), kidney petechiation + blood in pericardium and abdomen (1)	9
Miscellaneous lung changes Inflammation and consolidation of lungs with pleural haemorrhage (1), patchy lungs (2), stippled lungs (1), mottled lungs (4), abnormal lungs (1), blotchy lungs (1), congested lungs (3), congested lungs and blood at nares (1), pneumonia (1), pulmonary oedema (1), pale and blotchy lungs (1), consolidated lungs + small spleen + fluid in abdomen(1)	18
CHANGES IN SPLEEN WITHOUT LIVER OR LUNG ABNORMALITIES Splenomegaly (2), mottled appearance (1), spot on spleen and pericardial fluid (1)	4
KIDNEY CHANGES WITHOUT LIVER, LUNG OR SPLENIC ABNORMALITIES Haemorrhagic kidney (1), Kidney Petechiation (1), Kidney petechiation + blood at nares (1)	3
MISCELLANEOUS CHANGES WITHOUT LIVER, LUNG, SPLENIC OR KIDNEY ABNORMALITIES Blood at nares (6), haemorrhagic fluid in abdomen (4), peritoneal fluid (1)	11
Total	185

*Note*: The gross post mortem examination was carried out by a veterinary practitioner not a trained pathologist

### Comparison of macroscopic and microscopic findings

The macroscopic appearance of the internal organs often did not match the histopathological changes. Gross liver abnormalities were only reported in 55 of 185 (30%) cases despite all 185 cases showing hepatocellular necrosis on microscopic examination. Gross lung changes were reported in 41 of 185 (22%) cases but no important histopathological abnormality was found in 30 of 41 (73%) of these cases, although agonal changes, such as alveolar collapse, congestion and alveolar oedema, were evident in 23 of 30 (73%). In the remaining 11of 41 (27%) cases that showed macroscopic lesions, significant histopathological changes in the lungs were seen. These changes included intra‐alveolar haemorrhage (11 of 30, 37%) and fibrinous exudate or fibrin thrombi in the pulmonary blood vessels (17of 30, 56%). Conversely, in the 144 cases that reported no macroscopic abnormality of the lungs, 22 of 144 (15%) showed intra‐alveolar haemorrhage on histological examination.

Gross abnormalities of the spleen were reported in only eight of 185 (4%) cases. Splenomegaly was recorded in seven of these but only four had histopathologic abnormalities; three showed diffuse congestion, one with haemorrhage and two with multifocal lymphocytolysis and the fourth showed cell karyorrhexis across the white pulp Mild lymphocytolysis with fibrin in red pulp was seen in a spleen that was described as mottled. No gross abnormality of the spleen was recorded in 177 of 185 (96%) cases but histopathological changes were apparent in 84 of 177 (47%) of these cases. The changes ranged from moderate congestion to haemorrhage, deposition of fibrin, lymphocytolysis and necrosis of lymphoid tissue.

Macroscopic abnormalities of the kidney were only reported in nine of 185 (5%) and petichiation was the reported change. Two of these cases showed no significant microscopic changes and seven showed a variety of histopathological changes (*e.g*. focal interstitial infiltration and fibroplasia, multifocal interstitial nephritis, glomerular fibrin thrombi, glomerular congestion). Histopathological changes were diagnosed in the 157 of 176 (89%) cases in which no macroscopic abnormality was seen in the kidneys. These histopathological changes included glomerular thrombosis in 98 of 157 (62%) cases and/or congestion in 82 of 157 (52%). Evidence of focal interstitial inflammatory infiltrates, fibrosis or mineralisation were seen in 12 of 157 cases (8%).

Myocardial haemorrhages were reported as a macroscopic change in two of 185 (1%) cases and confirmed by histopathology in one; the other showed no heart disease. Conversely, myocardial haemorrhages were evident microscopically in eight cases in which macroscopic changes were not reported. Blood at the nares was seen in 12 of 185 (6%) cases, of which eight showed intra‐alveolar haemorrhage on histopathological examination of the lungs. Evidence of concurrent disease was found on gross examination and histopathology in 34 of 185 (18%) rabbits that died from RHD. Details are shown in Table 3.

**Table 3:** Concurrent diseases in 34 rabbits of 185 cases with histopathological features of rabbit haemorrhagic disease

Concurrent disease	Number
Reproductive tract disease	
Post‐spay adhesions between vaginal stump and bladder or bowel	2
Uterine tumours	3 (one in a spayed rabbit)
Respiratory disease	
Bronchopneumonia	1
Pulmonary histiocytosis	2
Heart disease	
Cardiomyopathy	4
Mild multifocal fibrosis	6
Mild multifocal mineralisation	1
Renal disease	
Interstitial fibrosis	4
Cysts in renal medulla	1
Intestinal disease	
Mural abscess	1
Intestinal obstruction	1
Pyloric hypertrophy	3
Lymphoplasmacytic infiltration	1
Liver disease	
Hepatic coccidiosis	4
Total	34

## DISCUSSION

We found the previously reported clinical presentations of peracute, acute and chronic forms of RHD difficult to differentiate because the criterion of sudden or unexpected death meant that the case histories did not provide a comprehensive description of the clinical features. Most of the rabbits (143 of 185) were dead or dying at the time of presentation. This contrasts with laboratory investigations in which rabbits are observed closely after receiving an infective dose of RHD virus. In laboratory investigations, pyrexia (<40°C) is a feature of RHD with hypothermia occurring just before death (Neimanis *et al*. 2018, Le Minor *et al*. 2019). Body temperature was rarely included in the case histories in our investigation, perhaps because it was not recorded, or because the rabbit was collapsed or already dead at the time of presentation. Clinically chronic cases were not identified in our investigation, although icterus was reported in five of 185 (2.7%) cases. Chronic cases can survive infection so they would not be included in an investigation of sudden or unexpected death. The terms peracute, acute and chronic were also used by the histopathologists to categorise histopathological changes, especially in the liver but in this context the terms did not necessarily reflect the clinical picture. For example, it was possible for a rabbit to die suddenly and show peracute widespread hepatocellular necrosis or show more chronic changes such as inflammation or fibroplasia (Fig. 4).

Death of additional rabbits in the household was recorded in 65% cases. There are only a few conditions that will cause multiple deaths in pet rabbits and histopathology can differentiate RHD from other causes. In juvenile rabbits, infectious disease, especially enteric disease, can kill multiple individuals and was the cause of death in eight of the rabbits in this study in which histopathology and PCR testing ruled out RHD (Part 2). Poisoning is a concern for many owners of rabbits that die suddenly, especially if there are multiple deaths in the same household. There are no plants that cause acute hepatic necrosis in rabbits and, although aflatoxins (Baker & Green 1987; Krishna et al.1991), copper toxicity (Ramirez *et al*. 2013) and some compounds such as norepinephrine (Lee *et al*. 1987) can cause liver necrosis, these conditions are unlikely in rabbits kept as pets and the pattern of hepatic necrosis differs from RHD. Heatstroke is another differential diagnosis for multiple deaths with hepatic necrosis but the circumstances surrounding the deaths are likely to suggest the diagnosis. Like RHD, the *post mortem* findings in cases of heatstroke include pulmonary haemorrhage, necrosis of the liver and signs of DIC such as fibrin thrombi in small blood vessels (Palmiere & Mangin 2013). *Post mortem* examination shortly after death is required to see these signs as raised body temperature favours autolysis, which is rapid in rabbit carcases.

In this investigation, RHD could not be diagnosed from gross *post mortem* examination alone; although the described macroscopic changes of RHD were apparent in some cases, there was no consistent pattern (Table 2). This agrees with OIE (2019b) that describes the gross pathological lesions as variable and possibly subtle. In 42% rabbits that died from RHD, no abnormality at all was reported on gross *post mortem* examination. It should be borne in mind that the *post mortem* examinations were performed by veterinary practitioners and gross lesions may have been reported in a higher proportion of cases if the macroscopic examination had been performed by a veterinary pathologist. In many instances, the macroscopic appearance of the organ did not match the microscopic appearance. Again, this may have been due to the inexperienced eye of a practitioner, although some causes would be readily apparent, such as neoplasia, gastric or intestinal rupture, peritonitis, gastric dilation and intestinal obstruction, liver lobe torsion or ureteral obstruction and hydronephropathy. Our previous experience is that there is marked autolysis in rabbit tissues examined 12 to 24 hours after death, even if the carcase has been chilled in a refrigerator. In this study, *post mortem* examination within 6 hours of death reduced the confounding effects of autolysis and enabled a more accurate assessment of both macroscopic and microscopic lesions.

Although a diagnosis of RHD could not be made from the macroscopic organ appearance, it could be made by histopathology because of the characteristic pattern of lesions consistent with RHD. There were two cases in this series of 300 rabbits in which histopathology was suspicious for, but not characteristic of, RHD. In the first case, the liver was affected by hepatic coccidiosis, which could have complicated a diagnosis of RHD (Fig. 6). In this case, the PCR results were equivocal (Part 2). The second suspicious case showed no histopathological features of RHD on initial examination, but areas of hepatocellular necrosis and glomerular thrombosis were eventually evident when the case was reviewed. This case also showed equivocal PCR results. It is probable that this rabbit died so quickly from RHD that microscopic changes did not have time to become widespread and were therefore not evident throughout the tissue. Cells that suffer a rapid onset terminal insult, such as infection by RHDV, will exhibit a time lag between cell injury or death and the appearance of necrosis or apoptosis that can be seen using light microscopy. It is suggested that this takes a minimum of 4 hours (Kumar 2010).

Although histopathology is often diagnostic for RHD, it does not always feature highly in recommended diagnostic procedures for RHD. More emphasis is placed on PCR testing (Rocchi & Dagleish 2018), which is regrettable as histopathology can yield valuable information about other causes of death and concurrent disease (Table 3). For example, sepsis is a major differential diagnosis for RHD because death is sudden and often accompanied by hepatic necrosis and DIC. In this study, sepsis was the cause of death in nine rabbits. The diagnosis was made from the pattern of hepatocellular necrosis, which is different from RHD (Fig. 7). Although sepsis was not confirmed by bacterial culture in these cases, there were microscopic signs of generalised bacteraemia in other organs, such as the lung.

Our conclusion is that a presumptive diagnosis of RHD can be made from a history of sudden deaths in a household, especially if the rabbits are unvaccinated. Before death, clinical signs are variable. *post mortem* examination by the veterinary practitioner is valuable: internal organs can be inspected and tissue samples can be collected for histopathological examination. There may or may not be macroscopic evidence of RHD, such as hepatomegaly, splenomegaly or haemorrhages but a diagnosis of RHD can be made from the histopathological findings, characterised by hepatocellular necrosis, often accompanied by glomerular thrombosis. A PCR test for RHDV1 or RHDV2 is necessary to identify the genotype. If an owner wishes to know whether their rabbit died from RHD, PCR on liver tissue is all that is necessary although occasional false negatives can occur (Part 2). If an owner wishes to know why their rabbit died, *post mortem* examination with histopathology is essential as it provides additional information about other causes of death.
